# Spousal Concordance in Overweight and Obesity among Indian Couples: A Nationwide Analysis of Socioeconomic and Dietary Determinants

**DOI:** 10.1016/j.cdnut.2025.107489

**Published:** 2025-06-21

**Authors:** Prashant Kumar Singh, Lucky Singh, Shashi Kala Saroj, Chandan Kumar, Shekhar Kashyap, Shalini Singh

**Affiliations:** 1 Division of Preventive Oncology and Population Health, ICMR-National Institute of Cancer Prevention and Research, Noida, Uttar Pradesh, India; 2 Faculty of Medical Research, The Academy of Scientific and Innovative Research (AcSIR), New Delhi, India; 3 Indian Council of Medical Research (ICMR), New Delhi, India; 4 Faculty of Mathematical and Information Sciences, Academy of Scientific and Innovative Research (AcSIR), New Delhi, India; 5 Department of Policy and Management Studies, TERI School of Advanced Studies (TERI SAS), New Delhi, India; 6 Emerson Centre of Excellence for Sustainability Studies, TERI SAS, New Delhi, India; 7 Interventional Cardiologist, New Delhi, India; 8 ICMR-National Institute of Cancer Prevention and Research, Noida, Uttar Pradesh, India

**Keywords:** spousal concordance, overweight, obesity, Asian-BMI, covariates, NFHS, India

## Abstract

**Objective:**

This study examines the level and determinants of spousal concordance in overweight/obesity among married couples in India.

**Background:**

The rising prevalence of overweight/obesity is a growing public health concern globally. While spousal concordance in obesity is recognized in developed countries, less is known about this phenomenon in low- and middle-income countries. Understanding the shared risk factors within couples is crucial for effective intervention.

**Methods:**

We analyzed data from 52,737 married couples using the nationally representative National Family Health Survey (NFHS)-5 (2019–2021). This study used the Asian body mass index (BMI) (in kg/m^2^) cutoff for overweight/obesity (≥23.0) concordance. Descriptive statistics were used to assess sociodemographic characteristics. The log-binomial regression model was used to estimate the adjusted risk ratio (ARR) of spousal concordance in overweight/obesity.

**Results:**

Overall, 27.4% of couples exhibited concordance for overweight or obesity. Concordance was more prevalent among couples belonging to the richest wealth quintile (47.6%), residing in urban areas (38.4%), living in nuclear families (28.9%), sharing similar age (28.8%) or higher (28.9%), or having similar education levels (31.4%). Higher concordance was also associated with couples not engaged in paid work (33.9%) and those who frequently used media (newspapers: 39.6%, television: 32.8%) or consumed nonvegetarian foods weekly—eggs (30.7%), chicken (29.9%). Geographically, the highest concordance was observed in the southern (37.2%) and northern (33.5%) regions, with Kerala, Jammu and Kashmir, Manipur, Delhi, Goa, Tamil Nadu, and Punjab reporting the highest state-level prevalence (≥42%). Multivariable analysis showed significantly increased risk of spousal overweight/obesity concordance among couples in the richest wealth quintile (ARR = 4.311; 95% CI: 3.757, 4.947), urban areas (ARR = 1.085; 95% CI: 1.016, 1.159), other religious groups (ARR = 1.185; 95% CI: 1.089, 1.291). Regular consumption of eggs (14%), fish (25%), chicken (9%), fried foods (6%), and alcohol (98%) were also linked to higher concordance.

**Conclusions:**

Spousal concordance in overweight/obesity is strongly influenced by shared socioeconomic status, lifestyle behaviors, and dietary patterns. Couples in urban, affluent, media-exposed, and nonvegetarian households are particularly at risk. Public health strategies should prioritize couple-based interventions, especially among high-risk subgroups, to curb the dual burden of overweight/obesity and associated chronic diseases.

## Introduction

The rise in overweight/obesity prevalance worldwide is an emerging threat to human well-being [1]. In 2022, ∼43% of the global adult population (2.5 billion people) was overweight [with a BMI (in kg/m^2^) ≥25], and among them, 16% of the total population (890 million adults) were obese (BMI ≥ 30) [2]. In India, nearly one-fourth of women (24.0%) and men (22.9%) aged 15–49 years were overweight/obese in 2021, with projections estimating this will rise to 27.4% (24.5%–30.6%) and 30.5% (27.4%–34.4%) women and men, respectively, by 2040 [3]. Region-specific analyses also indicate that the Asian population exhibits more significant overweight/obesity prevalence [4]. According to the Global Burden of Disease report, overweight/obesity is among the top five risk factors for noncommunicable diseases such as cardiovascular disease, stroke, diabetes, obstructive sleep apnea, and cancer, resulting in 160 million years of healthy life lost globally [5].

Overweight/obesity should not be viewed in isolation, as individuals exist within households or families with shared environments [6]. Spousal relationships are influenced by individual factors such as individual’s age, race, education, socioeconomic status, food choices, physical and mental health, family type, and life goals [7]. Available evidence suggests that marriage positively impacts an individual’s physical, mental, and emotional health due to changes in daily routine, food habits, and healthcare practices [8]. This is the result of nurture within a specific period. As per the National Family Health Survey (NFHS) conducted during 2019–2021, married women (29%) and men (29.0%) in India were more likely to be overweight and obese than unmarried women (8.2%) and men (13.4%) [9]. Another study has shown that married individuals are 88% more likely to become obese compared to unmarried, divorced, and widowed individuals [10]. Similarly, the Framingham Heart Study found that if one spouse became obese, the other spouse's likelihood of obesity increased by 37% [11].

Regarding the determinants of overweight/obesity, numerous studies have documented the positive association of a nonvegetarian diet (e.g., fish, chicken, eggs, and red meat) [12,13], saturated fats, or partially hydrogenated oils (trans fats) with elevated LDL concentrations and related diseases such as insulin resistance, obesity, and other metabolic disorders [14,15]. Substance use, including tobacco and alcohol, is also recognized as the major risk factors for multiple morbidities, including overweight/obesity [16,17]. However, confounding factors such as physical activity levels, socioeconomic status, age, and healthy behavioral practices strongly influence the health of couples [18,19].

The phenomenon of body mass index (BMI) similarity between spouses is termed spousal concordance of overweight or obesity, also known as co-overweight or co-obesity [20]. Spousal concordance has been measured in various ways [21]; for instance, studies suggest that 29% of couples gain more weight post-marriage and develop a higher risk of noncommunicable diseases, especially those who marry later in life [[22], [23], [24]]. While some studies have explored spousal dynamics and reciprocal associations related to health or health behaviors, thereby highlighting spousal concordance in health-related measures [18,25], previous research has lacked comprehensive analyses of spousal concordance in overweight/obesity, often focusing instead on individual prevalence rates and associated risk factors [26,27]. Furthermore, spousal concordance in overweight/obesity has not been extensively examined in low- and middle-income countries, particularly in India, which is one of the most populated countries with a substantial working-age population [28]. Addressing this knowledge gap is crucial; therefore, this study aimed to examine the level of spousal concordance in overweight/obesity and to assess the associated risks related to various background characteristics of married couples in India using large, cross-sectional population-based survey data.

## Methods

### Data

This study used the couple data from the fifth round of the Indian National Family Health Survey (NFHS)-5 (2019-2021) [9]. The survey collected information from 6,36,699 households and 57,693 couples, with women aged 15 to 49 y and men aged 15 to 54 y, across the country. Employing a stratified two-stage sampling design using probability proportional to size, NFHS-5 collected data from rural and urban households across all 28 states and 8 union territories of India [29]. Sample weights were applied to ensure that the data were representative of the total population [25]. The dataset, which is publicly available, was accessed from: https://dhsprogram.com/what-we-do/survey/survey-display-541.cfm. The research, conducted using NFHS data, adhered to established ethical guidelines and regulations. The NFHS data are publicly available and anonymized, provided by the International Institute for Population Sciences and the Ministry of Health and Family Welfare, Government of India. As such, the study did not involve direct interaction with human subjects or the collection of primary data, thus exempting it from the need for a formal ethics committee review.

### Selection of study participants

**FIGURE 1:**
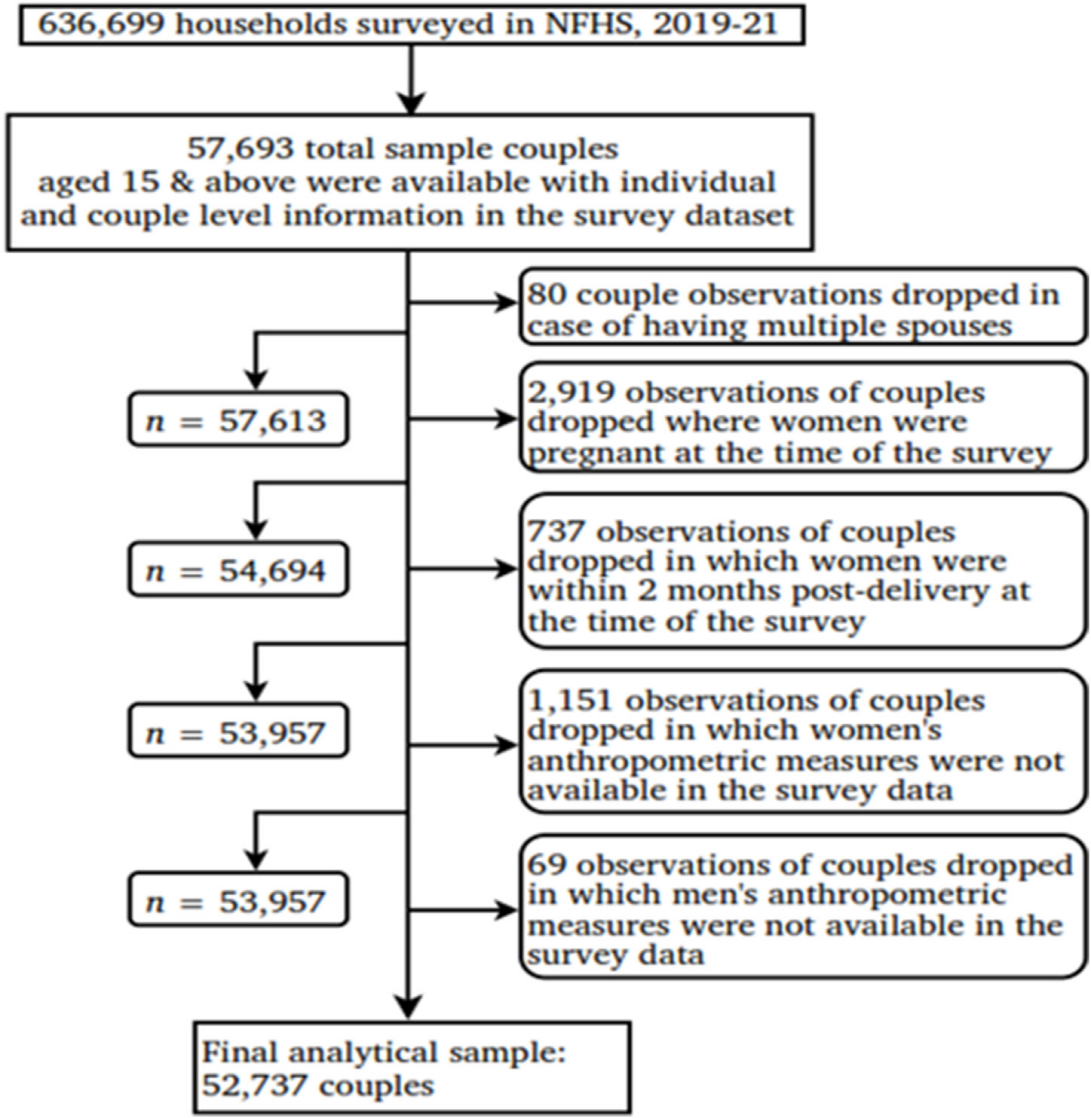
Flow diagram of selection for the study participants.

The data for this study were derieved from the couples’ file within the NFHS-5 dataset. To include key confounding variables such as social group, religion, region, and men’s BMI, both the household and person files were used. The final sample was selected by excluding irrelevant cases based on multiple criteria. From the initial 57,693 sample couples aged 15–54 y, 80 couples were excluded due to polygamous unions, and 2,919 sample couples were dropped due to wife's pregnancy at the time of the survey. Additionally, 737 sample couples were excluded due to the wife being within two months postdpartum during the survey. Further exclusions included 1,151 and 69 sample couples due to missing anthropometric details for women and men, respectively. Thus, the final sample for analysis consisted of 52,737 married couples. A detailed description of the sample selection process is presented graphically in Figure 1. Weighted analyses were conducted across the states to account for survey design and ensure representativeness.

### Outcome variable

The overweight/obesity was determined following the BMI cutoffs recommended for the Asian population, calculated as the ratio of weight (in kilograms) to height (in meters squared) for individuals. For this study, couples with a BMI ≥23.0 were categorized as couples with overweight/obesity and those with <23.0 as not overweight/obese couples. Married couples exhibiting concordance in overweight/obesity were coded as “1” (concordant couples), while couples with discordance were coded as “0” [9].

### Analytical strategy

Descriptive statistics (proportions) were used to describe the pattern of the spousal concordance in overweight/obesity across various socioeconomic, demographic, and nutritional covariates. The χ^2^ test was employed to assess the statistical significance of associations between concordance in overweight/obese married couples and covariates. Risk ratios were estimated to quantify the association between spousal concordance in overweight/obesity and the couples’ background characteristics using a generalized linear model (*glm*) with a binomial family and logit link function. The equation is represented as follows:

$$\log\left(P\left[Y_{i}=1|D_{i}=\left(X_{i,}C_{i}\right)\right]\right)=\beta_{0}+\beta_{1}X_{i}+\beta_{2}C_{1i}+\ldots +B_{n}C_{pi}$$

$$=\left(\beta D_{i}\right)$$

where, *log* denotes the natural logarithm; overweight/ obesity is a binary outcome variable (*Y*) coded as 1 for Yes and 0 for No; *X* is the group of exposure variables (e.g., couple's type of food consumption, smoking status, smokeless tobacco (SLT) use, and alcohol consumption); and *C* is a vector of confounders including couple's household wealth quintile, age-difference, mass-media exposure, social group, religion, place of residence, and region. The robustness of the model was also assessed using ARRs derived from GLM log-binomial regression models [30,31].

Covariates included in the final model were: 1) consumption of fried and non-vegetarian food at least a week (no, egg, fish, chicken, and fried food); *2*) SLT use (neither spouse uses, both spouses use, and one spouse uses); *3*) alcohol consumption (neither spouse consume, both spouses consume, and one spouses consumes); *4*) household wealth quintile (poorest, poorer, middle, richer, and richest); *5*) religion (Hindu, Muslim, and others); and *6*) place of residence (rural and urban). A detailed description of the covariates is provided in Table 1. The analysis was carried out using the *glm* command with *family(binomial*) and *link(log)* options in STATA software version 15 [32].

**TABLE 1:** Sample characteristics of the couples, National Family Health Survey (2019–2021), India.

Background characteristics	*n*	%
Place of residence		
Rural	39,833	67.3
Urban	12,904	32.7
Household wealth quintile		
Poorest	10,754	17.6
Poorer	11,687	19.8
Middle	11,218	21.6
Richer	10,251	21.9
Richest	8827	19.1
Religion		
Hindu	40,322	79.9
Muslim	6030	15.1
Others	6385	5.0
Social group		
Scheduled castes	10,157	21.1
Scheduled tribes	10,532	9.8
Other backward class	19,693	39.9
Others	12,355	29.3
Type of household		
Nuclear	27,058	50.5
Nonnuclear	25,679	49.5
Couple’s age difference (husband − wife)		
Wife older/no difference	3865	5.7
1–4 y	24,878	40.8
≥5 y	23,994	53.5
Couple’s education level		
No formal education	6137	11.0
Wife > husband	6957	15.2
Husband > wife	16,956	28.6
Similar level	22,687	45.2
Couple’s fried and nonvegetarian food consumption ≥1 wk		
No^1^	27,655	44.3
Egg	3295	7.1
Fish	3213	8.8
Chicken	7367	18.5
Fried food	11,207	21.3
Couple smoking tobacco		
Neither spouse smokes	43,576	84.5
Both spouse smoke	80	0.1
One spouse smokes	9081	15.5
Couple using smokeless tobacco		
Neither spouse uses	38,354	77.5
Both spouses use	1464	1.8
One spouse uses	12,919	20.7
Couple taking alcohol		
Neither spouse consumes	35,242	71.4
Both spouses consume	1103	0.9
One spouse consumes	16,392	27.6
Couple’s work profession		
Not working	1006	1.7
Similar profession	10,802	19.0
Dissimilar profession	40,929	79.3
Couple reading newspaper		
Not at all	22,346	38.6
Both read at least once a week	10,729	24.9
Any reads at least once a week	19,662	36.5
Couple watching television		
Not at all	6753	11.5
Both watch at least once a week	32,817	65.9
Any watches at least once a week	13,167	22.6
Region		
North	10,406	8.0
Central	11,529	10.3
East	8109	26.7
Northeast	7937	5.8
West	6273	23.9
South	8483	25.3
Total	52,737	100

1 Couples might be consuming nonvegetarian and fried food, but not on weekly basis.

## Results

### Characteristics of the selected married couples

Nearly 11% of couples had no formal education, whereas 45.2% had a similar level of education. Approximately 44.3% of couples did not consume nonvegetarian or fried foods, while 21.3% of couples consumed fried foods; 18.5% consumed chicken, 8.8% consumed fish, and 7.1% consumed eggs at least weekly. The use of any form of tobacco and alcohol by either spouse or both was reported by 38.1% and 28.5% of couples, respectively. Additionally, 79.3% of couples had dissimilar occupations, while 19% were engaged in the same profession.

Table 1 shows that the majority of couples (67.3%) lived in rural areas, belonged to the Hindu religion (79.9%), and were from other backward classes (39.9%). Over half of the wives were >5 y younger than their husbands (53.5%), whereas only 5.7% of surveyed couples reported being the same age or having wives older than their husbands.

### Spousal concordance in overweight/obesity

Table 2 suggests that nearly 27.4% (95% CI: 26.5%, 28.3%) of couples in India had concordance in overweight/obesity, with significant variations across socioeconomic and household characteristics. Higher concordance was found in urban areas (38.4%; 95% CI: 36.3%, 40.5%) than that in rural areas (22.1%; 95% CI: 21.3%, 22.9%). Nearly half of the couples in the richest wealth quintile (47.6%; 95% CI: 44.9%, 50.4%) exhibited concordance in overweight/obesity, followed by those in the richer (35.5%; 95% CI: 33.9%, 37.2%) and middle (25.5%; 95% CI: 24.1%, 26.9%) wealth quintiles. Higher spousal concordance was also exhibited by couples belonging to other religious group (36.1%; 95% CI: 33.0%, 39.2%), other social group (31.6%; 95% CI: 29.8%, 33.5%), and other backward classes (29.1%; 95% CI: 28.0%, 30.3%).

**TABLE 2:** Proportion (%) of overweight/obese couples by covariates, National Family Health Survey (2019–2021), India.

Exposures and covariates	% (95% CI)	*p value*
Couple’s fried and nonvegetarian food consumption ≥1 wk		*<0.001*
No^1^	25.4 (24.1, 26.7)	
Egg	30.7 (27.5, 34.2)	
Fish	29.2 (26.2, 32.2)	
Chicken	29.9 (28.0, 31.9)	
Fried food	27.6 (25.9, 29.3)	
Couple smoking tobacco		*0.095*
Neither spouse smokes	27.7 (26.7, 28.7)	
Both spouses smoke	29.8 (16.4, 47.8)	
One spouse smokes	25.8 (23.9, 27.8)	
Couple using smokeless tobacco		*<0.001*
Neither spouse uses	29.3 (28.3, 30.3)	
Both spouses use	15.6 (12.6, 19.1)	
One spouse uses	21.3 (19.7, 23.0)	
Couple taking alcohol		*<0.001*
Neither spouse consumes	28.3 (27.2, 29.4)	
Both spouses consume	18.3 (14.3, 23.1)	
One spouse consumes	25.4 (23.9, 27.0)	
Household wealth quintile		*<0.001*
Poorest	10.2 (9.0, 11.5)	
Poorer	16.3 (15.1, 17.7)	
Middle	25.5 (24.1, 26.9)	
Richer	35.5 (33.9, 37.2)	
Richest	47.6 (44.9, 50.4)	
Religion		*<0.001*
Hindu	26.6 (25.6, 27.6)	
Muslim	28.8 (26.8, 30.9)	
Others	36.1 (33.0, 39.2)	*<0.001*
Social group		
Scheduled castes	23.3 (21.7, 25.1)	
Scheduled tribes	16.7 (14.5, 19.2)	
Other backward class	29.1 (28.0, 30.3)	
Others	31.6 (29.8, 33.5)	
Type of household		*<0.001*
Nuclear	28.9 (27.6, 30.1)	
Nonnuclear	25.9 (24.9, 27.0)	
Couple’s age difference (husband − wife)		*<0.001*
Wife older/no difference	28.8 (25.6, 32.2)	
1–4 y	25.3 (24.1, 26.4)	
≥5 y	28.9 (27.7, 30.1)	
Couple’s education level		*<0.001*
No formal education	17.7 (15.8, 19.9)	
Wife > husband	26.4 (24.5, 28.5)	
Husband > wife	25.4 (24.0, 26.8)	
Similar level	31.4 (30.1, 32.7)	
Couple’s work profession		*<0.001*
Not working	33.9 (26.6, 42.0)	
Similar profession	23.5 (22.0, 25.2)	
Dissimilar profession	28.2 (27.2, 29.2)	
Couple reading newspaper		*<0.001*
Not at all	19.1 (18.1, 20.2)	
Both read at least once a week	39.6 (37.5, 41.8)	
Any reads at least once a week	27.8 (26.5, 29.2)	
Couple watching television		*<0.001*
Not at all	12.7 (11.3, 14.3)	
Both watch at least once a week	32.8 (31.6, 34.0)	
Any watches at least once a week	19.2 (17.9, 20.5)	
Place of residence		*<0.001*
Rural	22.1 (21.3, 22.9)	
Urban	38.4 (36.3, 40.5)	
Region		*<0.001*
North	33.5 (32.2, 34.8)	
Central	21.7 (20.7, 22.8)	
East	19.4 (17.8, 21.1)	
Northeast	21.6 (20.0, 23.3)	
West	27.8 (25.5, 30.3)	
South	37.2 (35.3, 39.1)	
Total	27.4 (26.5, 28.3)	

1 Couples might be consuming nonvegetarian and fried food, but not on weekly basis.

Interestingly, nuclear families (28.9%; 95% CI: 27.6%, 30.1%), spouses of similar ages (28.8%; 95% CI: 25.6%, 32.25) and younger wives with >5-y age gap (28.9%; 95% CI: 27.7%, 30.1%); spouses with similar education levels (31.4%; 95% CI: 30.1%, 32.7%), and couples where neither spouse was employed (33.9%; 95% CI: 26.6%, 42.0%) showed comparatively higher concordance in overweight/obesity than their respective counterparts.

Additionally, couples who read newspapers (39.6%; 95% CI: 37.5%, 41.8%) or watched television (32.8%; 95% CI: 31.6%, 34.0%) at least once a week exhibited greater concordance in overweight/obesity compared to couples who did not read (19.1%; 95% CI: 18.1%, 20.2%) or watch (12.7%; 95% CI: 11.3%, 14.3%) at all. Conversely, couples who did not consume any form of tobacco (SLT: 29.3%; with 95% CI: 28.3%, 30.3%; and smoked: 27.7%; 95% CI: 26.7%, 28.7%) or consume alcohol (28.3%; 95% CI: 27.2%, 29.4%) at all exhibited higher overweight/obesity concordance compared to couples where one or both spouses used these substances. Additionally, 25.4% (95% CI: 24.1%, 26.7%) of couples who did not consume any type of nonvegetarian food (egg, fish, or chicken) or fried foods exhibited overweight/obesity concordance, compared to ∼30.7% (95% CI: 27.5%, 34.2%) of couples who consumed eggs, 29.9% (95% CI: 28.0%, 31.9%) who consumed chicken and fish (29.2%; 95% CI: 26.2%, 32.2%), and 27.6% who consumed fried foods (95% CI: 25.9%, 29.3%), at least once a week.

Regional variations revealed that nearly two-fifths (37.2%; 95% CI: 35.3%, 39.1%) of couples with overweight/obesity in southern India showed concordance, which was relatively higher than in other regions. Every third couple in northern India (33.5%; 95% CI: 32.2%, 34.8%) and every fourth couple in western India (27.8%; 95% CI: 25.5%, 30.3%) showed concordance in overweight/obesity. Lower concordance rates were observed among couples in eastern (19.4%; 95% CI: 17.8%, 21.1%) and northeastern India (21.6%; 95% CI: 20.0%, 23.3%).

**FIGURE 2:**
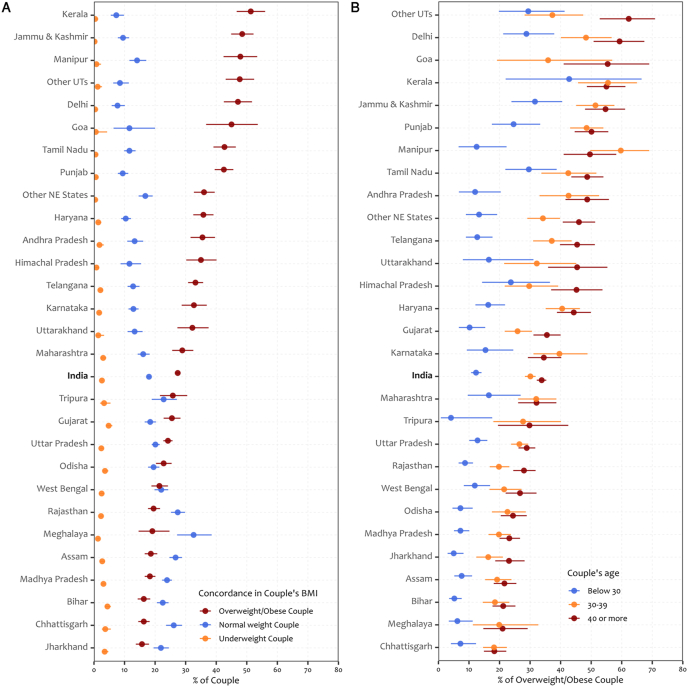
Concordance in the same category of couple’s BMI across states in India, 2019–2021. (A) Proportion (%) of overweight/obese, normal weight, and underweight couples across states. (B) Proportion (%) of couples with overweight and obesity by age group across states. NE, North-Eastern; UT, union territories.

Figure 2 shows the variation in overweight/obesity concordance among couples across Indian states, by stratified age groups. One-third of couples aged 40 y and above (33.8%; 95% CI: 32.2%, 35.3%) showed higher overweight/obesity concordance, followed by couples aged 30–39-y (30.1%; 95% CI: 28.3%, 31.8%) nationwide. However, across states, more than half of couples in Kerala (51.3%; 95% CI: 46.7%, 56.0%) and over two-fifths of couples in Jammu and Kashmir (48.5; 95% CI: 44.9%, 52.2%), Manipur (47.9%; 95% CI: 42.4%, 53.4%), Delhi (47.1%; 95% CI: 42.5%, 51.7%), Goa (45.0%; 95% CI: 36.7%, 53.6%), Tamil Nadu (42.7%; 95% CI: 39.1%, 46.4%), and Punjab (42.5%; 95% CI: 39.5%, 45.6%) exhibited overweight/obesity concordance.

Stratifying by age, more than half of couples aged 40 y and above showed concordance in overweight/obesity, particularly in Delhi (59.3%; 95% CI: 50.8%, 67.4%), Goa (55.4%; 95% CI: 41.0%, 69.0%), Kerala (55.0%; 95% CI: 48.6%, 61.2%), Jammu and Kashmir (54.7%; 95% CI: 48.0%, 61.1%), and Punjab (50.1%; 95% CI: 44.6%, 55.6%). Among couples aged 30–39y higher overweight/obesity concordance in Manipur (59.7%; 95% CI: 49.7%, 69.0%), Kerala (55.5%; 95% CI: 45.7%, 65.0%), Jammu and Kashmir (51.4%; 95% CI: 45.1%, 57.6%), Punjab (48.5%; 95% CI: 43.1%, 54.0%), and Delhi (48.3%; 95% CI: 40.1%, 56.7%). For couples aged 30 y and younger, higher overweight/obesity concordance was exhibited by those in Kerala (42.8%; 95% CI: 22.0%, 66.5%), Goa (37.0%; 95% CI: 5.9%, 84.7%), Jammu and Kashmir (31.6%; 95% CI: 23.9%, 40.5%), and Tamil Nadu (29.6%; 95% CI: 21.9%, 38.7%).

### Risk factors for spousal overweight/obesity concordance

Table 3 presents the ARRs for spousal concordance in overweight/obesity. The risk of overweight/obesity concordance was higher among couples who consumed fish (ARR: 1.251; 95% CI: 1.125, 1.391), eggs (ARR: 1.146; 95% CI: 1.022, 1.285), chicken (ARR: 1.097; 95% CI: 1.013, 1.189), or fried foods (ARR: 1.069; 95% CI: 1.005, 1.151) at least once a week, compared to non-consumers. While the point estimate suggests that couples where both consumed alcohol may have had a higher risk (ARR: 1.007; 95% CI: 0.804, 1.261), this result was not statistically significant. Likewise, SLT users had a lower risk of overweight/obesity concordance compared to non-users.

**TABLE 3:** Adjusted risk ratios for overweight/obese couples by covariates, National Family Health Survey (2019–2021), India.

Exposures and covariates	ARR (95% CI)	*P*
Couple’s fried and nonvegetarian food consumption ≥1 wk		
No^1^^,^^2^	1.000	
Egg	1.146 (1.022, 1.285)	*0.021*
Fish	1.251 (1.125, 1.391)	*<0.001*
Chicken	1.097 (1.013, 1.189)	*0.023*
Fried food	1.069 (1.005, 1.151)	*0.035*
Couple using smokeless tobacco		
Not at all^1^	1.000	
Both use	0.764 (0.609, 0.959)	*0.022*
Any spouse uses	0.893 (0.826, 0.965)	*0.004*
Couple taking alcohol		
Not at all^1^	1.000	
Both take	1.007 (0.804, 1.261)	*0.923*
Any spouse takes	0.984 (0.920, 1.052)	*0.056*
Household wealth quintile		
Poorest^1^	1.000	
Poorer	1.581 (1.365, 1.829)	*<0.001*
Middle	2.429 (2.132, 2.767)	*<0.001*
Richer	3.306 (2.895, 3.775)	*<0.001*
Richest	4.311 (3.757, 4.947)	*<0.001*
Religion		
Hindu^1^	1.000	
Muslim	1.084 (1.098, 1.178)	*0.045*
Others	1.185 (1.089, 1.290)	*<0.001*
Place of residence		
Rural^1^	1.000	
Urban	1.085 (1.016, 1.159)	*0.016*

1 Reference category.

2 Couples might be consuming nonvegetarian and fried food, but not on weekly basis.

Couples belonging to religious groups other than Hindu and Muslim had an 18.5% higher risk of overweight/obesity concordance (ARR: 1.185; 95% CI: 1.089, 1.290). Living in urban areas was associated with a 8.5% higher risk of overweight/obesity concordance compared to living in rural areas (ARR: 1.085; 95% CI: 1.016, 1.159). Strikingly, the risk of overweight/obesity concordance was remarkably 4.3 times higher among couples in the richest wealth quintile (ARR: 4.311; 95% CI: 3.757, 4.947) compared to those in the poorest wealth quintile. Couples in the richer (ARR: 3.306; 95% CI: 2.895, 3.775) and middle wealth quintiles (ARR: 2.429; 95% CI: 2.132, 2.767) also exhibited significantly higher risks of overweight/obesity concordance, at approximately 3 and 2 times greater than the poorest quintile, respectively. Even those in the poorer wealth quintile had a 58% (ARR: 1.581; 95% CI: 1.365, 1.829) higher risk than those in the poorest wealth quintile.

## Discussion

This study is among the first to examine the linkage between spousal concordance in overweight/obesity among married couples (15–49-y) in India. The study revealed intriguing patterns of concordance in overweight/obesity among married couples across different socioeconomic groups. In urban areas, over one-third of couples (38.4%) exhibited spousal concordance in overweight and obesity, whereas this concordance dropped to one-fifth of couples (22.1%) in rural areas. A substantially increased risk of similarity in couples’ overweight/obesity status was also seen with increasing wealth. Almost half of couples in the richest wealth quintile (47.6%) had a higher risk of overweight/obesity concordance compared to couples in the poorest wealth quintile.

This high spousal concordance in overweight/obesity, particularly among urban and wealthier couples, may be attributed to factors such as lower levels of physical activity, higher consumption of processed foods, and occupations primarily involving secondary and tertiary activities [33,34]. These lifestyle choices and work environments likely contribute to the similar overweight/obesity patterns observed among urban couples. On the contrary, spousal concordance overweight/obesity is less prevalent in rural areas, where a greater proportion of the population engages in primary activities that require more physical labor [33,35]. Factors such as a greater gender gap in marital relations, lower consumption of processed foods, and higher levels of household poverty may also contribute to the reduced similarity in overweight/obesity among rural couples [36,37]. These differences highlight the influence of occupational and lifestyle factors on health outcomes across different socioeconomic and geographic settings.

Furthermore, the study found that couples with similar levels of education exhibited higher concordance was observed among couples with no formal education [38]. This pattern suggests that similar educational backgrounds between spouses fosters more aligned habits in food consumption and physical activity [[39], [40], [41]].

Interestingly, the study also revealed that couples with only one working spouse showed higher concordance in overweight/obesity than those working in different or similar professions. Nonworking spouses, often homemakers, tend to have more sedentary lifestyles, look ways to spending extra time, such as entertaining themselves watching television at home, spend more time on meal preparation [42,43], are less inclined to engage in outdoor or physical activities, and invest less in costly, healthy food items [44,45].

Moreover, couples engaged in dissimilar professions showed higher concordance in overweight/obesity than those in similar professions, potentially due to their mismatched lifestyles that lead to unhealthy eating habits and a lack of synchronized time for physical activity and meal planning [46,47]. Additionally, couples who spent more time with mass media also risked higher concordance in overweight/obesity [48,49], likely due to reduced physical activity and exposure to advertisements promoting processed and ultra-processed foods, contributing to unhealthy eating habits [50,51].

The study also revealed that spousal concordance in overweight/obesity was higher among couples in nuclear families than in non-nuclear families [52]. Couples in nuclear families may consume more processed foods and have less time for physical activity, contributing to their higher similarity in overweight/obesity. In contrast, non-nuclear families might benefit from shared responsibilities and support systems that encourage healthier eating habits and more active lifestyles, leading to lower concordance in overweight/ obesity [53].

The findings also uncovered regional variations in spousal concordance in overweight/obesity. The southern region, relatively affluent in terms of human development indicators [2,54], exhibited higher concordance in overweight/obesity among couples than the northern region. In the northern region, most concordant overweight or obese couples were aged 40+-y [55]. In contrast, in the southern and western regions, overweight/obese couples were more prevalent among individuals younger than 40 y of age, highlighting the demographic and epidemiological transition of overweight/obesity across different states [56].

Consuming fried food or fish, chicken, or eggs weekly also increased the risk of overweight/obesity concordance. Fried food and processed meats are often high in saturated fat and sodium and tend to be more calorically dense than plant-based diets. Processed meat such as sausages, bacon, or deli meats often contain high sodium, preservatives, and additives to increase shelf life of the products, potentially contributing to weight gain and other health problems such as hypertension [12]. However, eggs and fish products are rich in lean protein, and varied eating patterns can aid in weight and nutritional management [57,58]. Overall, dietary pattern appears more important than simply whether or not someone consumes nonvegetarian foods or intoxicants; a balanced and varied nonvegetarian diet that includes plenty of fruits, vegetables, and whole grains may not be associated with an increased risk of overweight/obesity [59,60].

Regional differences also point to varying cultural and dietary preferences, with certain regions having predominant high-calorie food choices. High human development and per capita income in southern, western, and some northern states are linked to dietary shifts—from traditional, home-cooked diets to energy-dense, processed, and convenience foods [61]. For instance, in Kerala and Tamil Nadu, there has been a documented increase in household spending on processed and non-home-cooked foods over the last two decades, as per the National Sample Survey and Comprehensive National Nutrition Survey [62,63]. Additionally, these regions have relatively more urbanized populations, which shape dietary habits and sedentary lifestyles, contributing to the high prevalence of overweight/obesity among couples [42].

Assortative mating practices, which includes marriages between individuals of similar socioeconomic and demographic characteristics (education, age, region, religion, or caste) are also common in India [64,65]. This may contribute to the similarity in the health outcomes among couples, as they share the same background characteristics [66]. Hence, the findings of the study highlight the complex interplay between socioeconomic status and spousal overweight/obesity concordance, demonstrating how lifestyle and economic conditions can shape health outcomes within married couples.

A few limitations of this study include the cross-sectional data, making causal inference challenging as it is hard to establish whether couples’ concordance in overweight/obesity may have taken place post-marriage or continued from pre-marriage. The study has excluded pregnant and postpartum women, polygamous unions, and individuals with missing anthropometric data, which may lead to selection bias [67,68]. The impact of such bias, however, is likely minimal based on similarity in sociodemographic characteristics between the included study population and the excluded. Additionally, data on carbohydrate consumption and physical activity were not available for analysis. Further, the study did not include information on meal frequency, meal type, and other health-related behavior factors that may influence concordance in overweight/obesity among couples.

In conclusion, this study found that spousal concordance in overweight/obesity is notably higher among urban, affluent couples older than 40 y of age, particularly those who are non-working or engaged in dissimilar professions; those who consume fried food, eggs, fish, or chicken on weekly basis; and reside in nuclear families, especially in southern and northern regions. To address the global burden of noncommunicable diseases and achieve the Sustainable Development Goals by 2030 [69], targeted interventions are needed. Promoting healthy lifestyles, improving dietary habits, and increasing physical activity through awareness campaigns and targeted couple interventions can benefit both partners and contribute to overall public health improvements. Marriage can be the starting point in the exploration of the impact of social networks on the spread of obesity. It can be used to understand the impact of social networks on other non-communicable diseases and in low- and middle-income countries.

## Author contributions

The authors’ responsibilities were as follows – PKS, LS, SS: conceived and designed the study; PKS, LS, CK: were responsible for study methodology, formal analysis, and data visualization; SKS, PKS: wrote the original draft and edited the draft; LS, PKS, CK, SK, SS: supervised the draft and reviewed and edited the manuscript; LS, PKS, SKS, CK: had full access to the data in the study and took responsibility for the integrity of the data and the accuracy of the data analysis as well as the decision to submit for publication; and all authors: reviewed and approved the final version of the submitted manuscript.

## Data availability statement

Deidentified couples’ data will be available upon request to the corresponding author.

## Funding

The authors declare that the research was conducted in the absence of any commercial or financial relationships that could be construed as a potential conflict of interest. Funding sources were not involved in the study design, data collection, analysis and interpretation, writing of the manuscript, or decision to submit the article for publication.

## Conflict of interest

The authors report no conflicts of interest.

## References

[1] GBD 2017 Diet Collaborators (2019). Health effects of dietary risks in 195 countries, 1990–2017: a systematic analysis for the Global Burden of Disease Study 2017. Lancet.

[2] GBD 2019 Diseases and Injuries Collaborators (2020). Global burden of 369 diseases and injuries in 204 countries and territories, 1990–2019: a systematic analysis for the Global Burden of Disease Study 2019. Lancet.

[3] Luhar S., Timæus I.M., Jones R., Cunningham S., Patel S.A., Kinra S. (2020). Forecasting the prevalence of overweight and obesity in India to 2040. PLOS One.

[4] Misra A. (2015). Ethnic-specific criteria for classification of body mass index: a perspective for Asian Indians and American Diabetes Association Position Statement, Diabetes Technol. Ther.

[5] Felisbino-Mendes M.S., Cousin E., Malta D.C., Machado Í.E., Ribeiro A.L.P., Duncan B.B. (2020). The burden of non-communicable diseases attributable to high BMI in Brazil, 1990-2017: Findings from the Global Burden of Disease Study. Popul. Health Metr..

[6] Brahmanandam N., Nagarajan R. (2021). Impact of change in household environment condition on morbidity in India: evidence from longitudinal data. PLOS One.

[7] Shivani B.S.V. (2024). An effect of marital compatibility among married couples. Int. J. Indian Psychol..

[8] Nyberg S.T., Batty G.D., Pentti J., Virtanen M., Alfredsson L., Fransson E.I. (2018). Obesity and loss of disease-free years owing to major non-communicable diseases: a multicohort study. Lancet Public Health.

[9] International Institute for Population Sciences (IIPS), ICF (2021). National family health survey (NFHS-5) India 2019-21, Demographic Health Survey [Internet].

[10] Nikolic Turnic T., Jakovljevic V., Strizhkova Z., Polukhin N., Ryaboy D., Kartashova M. (2024). The association between marital status and obesity: a systematic review and meta-analysis. Diseases.

[11] Christakis N.A., Fowler J.H. (2007). The spread of obesity in a large social network over 32 years. N. Engl. J. Med..

[12] Schlesinger S., Neuenschwander M., Schwedhelm C., Hoffmann G., Bechthold A., Boeing H. (2019). Food groups and risk of overweight, obesity, and weight gain: a systematic review and dose-response meta-analysis of prospective studies. Adv. Nutr..

[13] Drouin-Chartier J.P., Chen S., Li Y., Schwab A.L., Stampfer M.J., Sacks F.M. (2020). Egg consumption and risk of cardiovascular disease: three large prospective US cohort studies, systematic review, and updated meta-analysis. BMJ.

[14] Ruuth M., Lahelma M., Luukkonen P.K., Lorey M.B., Qadri S., Sädevirta S. (2021). Overfeeding saturated fat increases LDL (low-density lipoprotein) aggregation susceptibility while overfeeding unsaturated fat decreases proteoglycan-binding of lipoproteins. Arterioscler. Thromb. Vasc. Biol..

[15] Engel S., Tholstrup T. (2015). Butter increased total and LDL cholesterol compared with olive oil but resulted in higher HDL cholesterol compared with a habitual diet. Am. J. Clin. Nutr..

[16] Saxena S., Singh P.K., Singh L., Kashyap S., Singh S. (2023). Smokeless tobacco use and public health nutrition: a global systematic review. Public Health Nutr.

[17] (January 2020). WHO Framework Convention on Tobacco Control, Indicator compendium for the global strategy to accelerate tobacco control.

[18] Weare A.R., Feng Z., McGrath N. (2023). The prevalence of hypertension and hypertension control among married Namibian couples. PLOS One.

[19] GBD Risk Factors Colloborators (2024). Global burden and strength of evidence for 88 risk factors in 204 countries and 811 subnational locations, 1990–2021: a systematic analysis for the Global Burden of Disease Study 2021. Lancet.

[20] Oktaria V., Mahendradhata Y. (2022). The health status of Indonesia’s provinces: the double burden of diseases and inequality gap. Lancet Glob. Health.

[21] Pauly T., Weber E., Hoppmann C.A., Gerstorf D., Scholz U. (2025). In it together: relationship transitions and couple concordance in health and well-being. Pers. Soc. Psychol. Bull..

[22] Baby J., Varghese J.S., Cyriac S., Narayan K.M.V., Kurpad A.V., Thomas T. (2021). Contribution of economic and nutritional context to overweight/obesity dynamics in Indian women from 1998 to 2016: a multilevel analysis of national survey data. BMJ Open.

[23] Tang F., Pan Y., Deng H. (2024). Effect of marriage on overweight and obesity: evidence from China. BMC Public Health.

[24] Quan S., Zhang H. (2024). The relationship between marriage and body mass index in China: evidence from the China Health and Nutrition Survey. Econ. Hum. Biol..

[25] Patel S.A., Dhillon P.K., Kondal D., Jeemon P., Kahol K., Manimunda S.P. (2017). Chronic disease concordance within Indian households: a cross-sectional study. PLOS Med.

[26] Verma M., Esht V., Alshehri M.M., Aljahni M., Chauhan K., Morsy W.E. (2023). Factors contributing to the change in overweight/obesity prevalence among Indian adults: a multivariate decomposition analysis of data from the National Family Health Surveys. Adv. Ther..

[27] Ramamoorthy T., Leburu S., Kulothungan V., Mathur P. (2022). Regional estimates of noncommunicable diseases associated risk factors among adults in India: results from National Noncommunicable Disease Monitoring Survey. BMC Public Health.

[28] Naciones Unidas (UN) (2024). http://www.un.org/development/desa/pd/.

[29] DuBois J.M., Iltis A.S. (2005). Research ethics.

[30] Roshchina Y., Roshchin S., Rozhkova K. (2022). Determinants of COVID-19 vaccine hesitancy and resistance in Russia. Vaccine.

[31] Mittinty M.N., Lynch J. (2023). Reflection on modern methods: Risk ratio regression—Simple concept yet complex computation. Int. J. Epidemiol..

[32] StataCorp (2021).

[33] GBD 2019 Risk Factors Collaborators (2020). Global burden of 87 risk factors in 204 countries and territories, 1990–2019: a systematic analysis for the Global Burden of Disease Study 2019. Lancet.

[34] Barua S. (2023). Spatial inequality and explaining the urban-rural gap in obesity in India: evidence from 2015–16 population-based survey. PLOS One.

[35] Sengupta P., Puri R. (2022). Gender pay gap in India: a reality and the way forward—an empirical approach using quantile regression technique. Stud. Microecon..

[36] Aiyar A., Rahman A., Pingali P. (2021). India’s rural transformation and rising obesity burden. World Dev.

[37] Templin T., Cravo Oliveira Hashiguchi T., Thomson B., Dieleman J., Bendavid E. (2019). The overweight and obesity transition from the wealthy to the poor in low-and middle-income countries: a survey of household data from 103 countries. PLOS Med.

[38] Bloch K.V., Klein C.H., de Souza e Silva N.A., da Rocha Nogueira R.A., Salis L.H.A. (2003). Socioeconomic aspects of spousal concordance for hypertension, obesity, and smoking in a community of Rio de Janeiro, Brazil, Arq. Bras. Cardiol..

[39] Niemi J. (2019). Promoting gender equality. Ius Gentium.

[40] Taddei C., Zhou B., Bixby H., Carrillo-Larco R.M., Danaei G., Jackson R.T. (2020). Repositioning of the global epicentre of non-optimal cholesterol. Nature.

[41] Mahmoud R., Kimonis V., Butler M.G. (2022). Genetics of obesity in humans: a clinical review. Int. J. Mol. Sci..

[42] Saboo B., Shah S., Talaviya P., Vyas C., Chandarana H., Nayak H. (2014). Prevalence of obesity and overweight in housewives and its relation with household activities and socio-economical status. J. Obes. Metab. Res..

[43] Sruthi K.G., John S.M., Marconi David S. (2023). Assessment of obesity in the Indian setting: a clinical review. Clin. Epidemiol. Glob. Health.

[44] Rao M., Afshin A., Singh G., Mozaffarian D. (2013). Do healthier foods and diet patterns cost more than less healthy options? A systematic review and meta-analysis. BMJ Open.

[45] Lewis N.A., Yoneda T. (2021). Within-couple personality concordance over time: the importance of personality synchrony for perceived spousal support. J. Gerontol. Ser. B Psychol. Sci. Soc. Sci..

[46] Contillo J.T. (2021).

[47] Apostolic Christian Counseling and Family Services (2016). Roles, responsibilities, and decision making in marriage roles. Apostolic Christian Counseling and Family Services.

[48] Abioye A.I., Hajifathalian K., Danaei G. (2013). Do mass media campaigns improve physical activity? a systematic review and meta-analysis. Arch Public Health.

[49] Pahari S., Acharya B., Chauhan H.S. (2017). Impact of mass media and socio demographic factors on physical exercises and food habits among adolescents in Pokhara sub metropolitan municipality, Nepal. Int. J. Med. Health Res..

[50] Fagerberg P., Langlet B., Oravsky A., Sandborg J., Löf M., Ioakimidis I. (2019). Ultra-processed food advertisements dominate the food advertising landscape in two Stockholm areas with low vs high socioeconomic status. Is it time for regulatory action?. BMC Public Health.

[51] Arrona-Cardoza P., Labonté K., Cisneros-Franco J.M., Nielsen D.E. (2023). The effects of food advertisements on food intake and neural activity: a systematic review and meta-analysis of recent experimental studies. Adv. Nutr..

[52] Purkayastha N., Dhillon P., Ali B., Hazarika J. (2024). Changing patterns of one-person and one-couple-only households in India. J. Popul. Ageing..

[53] Pillai R.K., Premaletha N., Saradamma R., Nair M.K.C., Savithriamma V.K., Soman S. (2022). Changing families and its effect on the health of family members in Kerala: a qualitative exploration. Clin. Epidemiol. Glob Health..

[54] Gouda J., Prusty R.K. (2014). Overweight and obesity among women by economic stratum in Urban India. J. Health Popul. Nutr..

[55] Lartey S.T., Si L., Otahal P., de Graaff B., Boateng G.O., Biritwum R.B. (2020). Annual transition probabilities of overweight and obesity in older adults: evidence from World Health Organization Study on global AGEing and adult health. Soc Sci Med.

[56] Jeong S., Cho II S. (2018). Concordance in the health behaviors of couples by age: a cross-sectional study. J. Prev. Med. Public Health.

[57] Puglisi M.J., Fernandez M.L. (2022). The health benefits of egg protein. Nutrients.

[58] Prajapati B.G. (2023). Impact of eggs protein on musculoskeletal health, Novel Tech. Nutr. Food Sci.

[59] Manchanda N., Kalra A., Arya M. (2022). Sustainable food consumption in india: present state, viability, barriers and possibilities. South Asian Res. J. Humanit. Soc. Sci..

[60] Samaddar A., Cuevas R.P., Custodio M.C., Ynion J., Ray C.A., Mohanty S.K. (2020). Capturing diversity and cultural drivers of food choice in eastern India. Int. J. Gastron. Food Sci.

[61] Dandona L., Dandona R., Kumar G.A., Shukla D.K., Paul V.K., Balakrishnan K. (2017). Nations within a nation: variations in epidemiological transition across the states of India, 1990–2016 in the Global Burden of Disease Study. Lancet.

[62] Radhakrishnan V., Varghese R.R. (February 9, 2024).

[63] (June 9, 2024). Kerala tops in share of spend on non-veg in food products: survey.

[64] Agarwal Goel P., Barua R. (2023). Female education, marital assortative mating, and dowry: theory and evidence from districts of India. J. Demogr. Econ..

[65] Mayo O., Nanjundiah V. (2024). Reflections on assortative mating, social stratification, and genetics. J. Genet..

[66] Borker G., Eeckhout J., Luke N., Minz S., Munshi K., Swaminathan S. (2022).

[67] Khalifa E., El-Sateh A., Zeeneldin M., Abdelghany A.M., Hosni M., Abdallah A. (2021). Effect of maternal BMI on labor outcomes in primigravida pregnant women. BMC Pregnancy Childbirth.

[68] Antonakou A., Papoutsis D., Tzavara C. (2018). Maternal obesity and its association with the mode of delivery and the neonatal outcome in induced labour: implications for midwifery practice. Eur. J. Midwifery..

[69] Independent Group of Scientists appointed by the Secretary-General, Global Sustainable Development Report 2023: Times of crisis, times of change: Science for accelerating transformations to sustainable development. New York: United Nations (2023). Available from: https://sdgs.un.org/sites/default/files/2023-09/FINAL%20GSDR%202023-Digital%20-110923_1.pdf. Date cited: 9 May 2025.

